# Hospital Account Keeping and the War: The Allocation of War Expenditure

**Published:** 1915-01-16

**Authors:** 


### HOSPITAL ACCOUNT KEEPING AND THE WAR.

### The Allocation of War Expenditure.

BY A HOSPITAL OFFICER.

In response to inquiries addressed to them, the three Funds have circulated a memorandum containing instructions as to the treatment in hospital accounts of expenditure incurred by reason of the war.

### The Funds' Memorandum.

The most important perhaps of these instructions is that where a salary or part salary is paid to an employee on military service, such payment should be treated as Extraordinary Expenditure. This is a course which would probably not have suggested itself to hospital secretaries acting on their own initiative, though there are obvious reasons why these unusual payments should not go to swell unduly the Ordinary Expenditure. From the remark in the memorandum that "salaries or wages paid to those who are doing the work of the employees so engaged will be charged to Ordinary Expenditure, and will, of course, form part of the cost per bed," it may be inferred that the ruling of the Funds has been inspired by the consideration that two salaries should not appear in respect of the same work. It may, however, be submitted that an employee on military duty is not always replaced by another officer, the work in some instances being carried on as far as possible by the remaining members of the staff. In such cases, to place the absent officer's salary to Extraordinary Expenditure will unduly decrease the Ordinary Expenditure, and thus disadvantageously affect the criterion by which the financial position of a hospital is judged. It would seem more equitable if the Funds' directions on this head were to apply only in cases where an employee on military service has been temporarily replaced by another at an approximately equal salary.

### Expenditure on Wounded Soldiers.

Amongst the other points dealt with in the same memorandum is that of expenditure incurred in the treatment of naval and military patients. This is to be charged to Ordinary Expenditure under the appropriate sub-heads. The same wording (putting "income" for "expenditure") is used in regard to "any moneys received or specially collected" in respect of the treatment of sailors and soldiers. It is interesting to note that the Funds do not specifically mention grants from public funds in this connection, and there may easily be some hesitation as to what is "the appropriate sub-head" for such grants. A certain latitude is, however, allowed by the remark that such expenditure and receipts may be separately stated if desired, "provided that they are included in the total of the sub-head."

A further point dealt with, it is to be observed, is in connection with institutions which have wholly or in part been taken over by the military or naval authorities. Beyond the remark that in such cases it would seem advisable to keep the accounts separate from those under the Uniform System, the Funds suspend judgment, and request that their advice may be immediately sought where such a case arises.

### What is a "Sub-head"?

Arising out of the memorandum from the three Funds on the question of expenditure and receipts in connection with the war, some hospital secretaries must be asking themselves what is really a "sub-head" in the Uniform System. It has been very generally held that a "sub-head" is the irreducible minimum, or, as it were, the unit. Thus, to take a simple case, "Patients' Payments" has been regarded as a main head, and "In-patients" and "Out-patients" as sub-heads. The Funds now say that payment in respect of soldiers (for example) should be "entered as Ordinary Income under the appropriate sub-head," or, if desired, "may be separately stated, provided it is included in the total of the sub-head, in accordance with paragraph 5, page 5" (of the Red Book).

The paragraph referred to states that "if it is desired to publish more details than the form provides for, they must be given so as to cast to one or other of the sub-heads." Now, it is only reasonable to infer that in the case of payments for soldiers the Funds wish such receipts, if shown separately, to cast to "Patients' Payments," which therefore would appear to be, in their opinion, a sub-head. It is true that identical wording in reference to this point is used in the Report of the Committee of Hospital Secretaries (October 6, 1906); but the construction which is generally placed on the term "sub-head" is plainly indicated in the Report of the Secretaries' Committee of 1891: "Few items appertaining to surgery and dispensary will be found, for the reason that the five sub-heads of this class are so descriptive that there can be little difficulty in the classification of items." Thus the instruction of the three Funds means either that in saying "sub-head" they refer to what is generally regarded as a "main head," or that they are prepared to sanction the sub-division of what has been termed hitherto the "irreducible minimum."

